# Mitochondrial DNA Mutations in Mutator Mice Confer Respiration Defects and B-Cell Lymphoma Development

**DOI:** 10.1371/journal.pone.0055789

**Published:** 2013-02-13

**Authors:** Takayuki Mito, Yoshiaki Kikkawa, Akinori Shimizu, Osamu Hashizume, Shun Katada, Hirotake Imanishi, Azusa Ota, Yukina Kato, Kazuto Nakada, Jun-Ichi Hayashi

**Affiliations:** 1 Faculty of Life and Environmental Sciences, University of Tsukuba, Tsukuba, Japan; 2 Mammalian Genetics Project, Tokyo Metropolitan Institute of Medical Science, Tokyo, Japan; University of Texas Health Science Center at San Antonio, United States of America

## Abstract

Mitochondrial DNA (mtDNA) mutator mice are proposed to express premature aging phenotypes including kyphosis and hair loss (alopecia) due to their carrying a nuclear-encoded mtDNA polymerase with a defective proofreading function, which causes accelerated accumulation of random mutations in mtDNA, resulting in expression of respiration defects. On the contrary, transmitochondrial mito-miceΔ carrying mtDNA with a large-scale deletion mutation (ΔmtDNA) also express respiration defects, but not express premature aging phenotypes. Here, we resolved this discrepancy by generating mtDNA mutator mice sharing the same C57BL/6J (B6J) nuclear background with that of mito-miceΔ. Expression patterns of premature aging phenotypes are very close, when we compared between homozygous mtDNA mutator mice carrying a B6J nuclear background and selected mito-miceΔ only carrying predominant amounts of ΔmtDNA, in their expression of significant respiration defects, kyphosis, and a short lifespan, but not the alopecia. Therefore, the apparent discrepancy in the presence and absence of premature aging phenotypes in mtDNA mutator mice and mito-miceΔ, respectively, is partly the result of differences in the nuclear background of mtDNA mutator mice and of the broad range of ΔmtDNA proportions of mito-miceΔ used in previous studies. We also provided direct evidence that mtDNA abnormalities in homozygous mtDNA mutator mice are responsible for respiration defects by demonstrating the co-transfer of mtDNA and respiration defects from mtDNA mutator mice into mtDNA-less (ρ^0^) mouse cells. Moreover, heterozygous mtDNA mutator mice had a normal lifespan, but frequently developed B-cell lymphoma, suggesting that the mtDNA abnormalities in heterozygous mutator mice are not sufficient to induce a short lifespan and aging phenotypes, but are able to contribute to the B-cell lymphoma development during their prolonged lifespan.

## Introduction

It has been hypothesized that pathogenic mtDNA mutations that induce significant mitochondrial respiration defects cause mitochondrial diseases [1], [2] and could also be involved in aging and age-associated disorders including tumor development [1]–[5]. This hypothesis is partly supported by studies in mtDNA mutator mice [6]–[8]: they possess a nuclear-encoded mtDNA polymerase with a defective proofreading function that leads to enhanced accumulation of random mutations in mtDNA with age, and the subsequent phenotypic expression of age-associated respiration defects and premature aging phenotypes, but not tumor development.

On the contrary, our previous studies [9], [10] showed that transmitochondrial mito-miceΔ carrying mtDNA with a large-scale deletion mutation (ΔmtDNA) expressed age-associated respiration defects, but not express the premature aging phenotypes. Similar results were obtained in other transmitochondrial mito-miceCOI^M^, which have an mtDNA point mutation in the *COI* gene [11]. Recently, we generated new transmitochondrial mito-miceND6^M^, which have an mtDNA point mutation in the *ND6* gene [12] that is derived from Lewis lung carcinomas, and confers respiration defects and overproduction of reactive oxygen species (ROS) [13]. Mito-miceND6^M^ did not express premature aging phenotypes, but were prone to B-cell lymphoma development [14]. Thus, it appears to be discrepant that premature aging phenotypes are exclusively observed in mtDNA mutator mice [6]–[8], but not in transmitochondrial mito-mice [9]–[11], [14], even though they all express mitochondrial respiration defects caused by mutated mtDNA.

This discrepancy may partly be the result of differences in the nuclear genetic background between mtDNA mutator mice and transmitochondrial mito-mice. It is also possible that the premature aging phenotypes found exclusively in mtDNA mutator mice are not caused by mutations in mtDNA, because inter-mitochondrial interaction and the resultant genetic complementation in mammalian mitochondria may prevent random mutations in mtDNA being expressed as respiration defects [10], [15], [16].

To clarify these issues, first we generated mtDNA mutator mice with the same C57BL/6J (B6J) nuclear genetic background as that of mito-miceΔ, and examined whether they still expressed respiration defects and premature aging phenotypes. We then transferred mtDNA from mtDNA mutator mice into mtDNA-less (ρ^0^) mouse cells and isolated transmitochondrial cybrids possessing mtDNA transferred from the mtDNA mutator mice, but not possessing defective mtDNA polymerase from the mtDNA mutator mice, and examined whether the resultant transmitochondrial cybrids expressed the expected respiration defects.

## Materials and Methods

### Mice

Inbred B6J mice generated by sibling mating more than 40 times were obtained from CLEA Japan. Mito-miceΔ were generated in our previous report [9]. Homo- and heterozygous mtDNA mutator mice were generated based on the procedures reported previously [6], [7] with a few modifications. We converted nucleotides 4460–4465 of the PolgA sequence from GACCGA to GCGCGC to introduce a D257A mutation in PolgA and create *BssH*II site for genotyping by PCR-RFLP method. Animal experiments were performed in accordance with protocols approved by the Experimental Animal Committee of the University of Tsukuba, Japan (Approval number: 12-295).

### Mouse Cell Lines and Cell Culture

Mouse B82 cells are fibrosarcomas derived from the L929 fibroblast cell line (C3H/An mouse strain) [17], and ρ^0^ B82 cells without mtDNA were obtained in our previous study [9]. Trans-mitochondrial cybrids were isolated by the fusion of the platelets from mtDNA mutator mice with ρ^0^ B82 cells by polyethylene glycol and subsequent selection that allows exclusive growth of the trans-mitochondrial cybrids (see Table S1). For isolation of immortalized 3T3 cells, MEFs in a 6-cm culture dish at a density of 3×10^5^ cells per dish were cultured using the 3T3 protocol [18], [19]. Briefly, 3 days after the cells had been plated at 3×10^5^ cells per dish, we trypsinized them, counted the total cell numbers, and then replated 3×10^5^ cells into 6-cm dishes. These processes were repeated until immortalized cells appeared. The mouse cells and cell lines were grown in DMEM (Sigma) containing 10% fetal calf serum, uridine (50 ng/ml), and pyruvate (0.1 mg/ml).

### Estimation of ΔmtDNA Proportion in Mito-Mice

The proportion of wild type mtDNA and ΔmtDNA was determined by a real-time PCR technique, as described previously [20].

### Analysis of COX Activity

Histochemical analyses for COX and SDH activity were carried out based on the procedures as described previously [21] using cryosections (10 µm thick) of cardiac muscles and renal tissues, and coverslips with growing 3T3 cells.

### Measurement of O_2_ Consumption Rates in Mouse Cell Lines

The rate of oxygen consumption was measured by trypsinizing cells, incubating the suspension in PBS, and recording oxygen consumption in a 2.0-ml polarographic cell at 37°C with a Clark-type oxygen electrode (Yellow Springs Instruments).

### Analysis of mtDNA Mutations

Total DNA was extracted from cells and tissues, and somatic mtDNA mutation load was determined by PCR, cloning, and sequencing, as described earlier (Ref.), using primers that specifically amplified a part of *COX1* gene (nucleotide pair 6,006–6,522) of mouse mtDNA. PCR products were subcloned into the pTA2 T-vector using by TArget Clone Plus Kit (TOYOBO, Osaka, Japan). Fifty plasmids were randomly selected from the each sample, were isolated using DirectPrep 96 MiniPrep Kit (Qiagen, Valencia, CA) and sequenced with M13 forward and reverse primers using BigDye Terminator Kit (Life Technologies, Grand Island, NY) on an Applied Biosystems 3130xl Genetic Analyzer. Sequences were assembled and edited in GENETYX ver10 (GENETYX Corporation, Tokyo, Japan).

### Southern-blot Analysis

Total DNA (5 µg and 3 µg) extracted from cells and tissues was digested with the restriction endonuclease *Xho*I. Restriction fragments were separated in 1.0% agarose gel, transferred to Hybond N^+^ membrane (GE Healthcare Lifesciences) and hybridized with alkaline phosphatase-labelled mouse mtDNA probes. Probe-bound alkaline phosphatase was used to catalyse light production by enzymatic decomposition of CDP-Star Detection Reagent (GE Healthcare Lifesciences). Chemiluminescences of fragments were measured with a bioimaging analyser, EZ-Capture ST (ATTO).

### Lactate and Glucose Measurement

To determine blood lactate and glucose concentrations, blood was collected from the tail veins of mice. Lactate and glucose concentrations were measured with an automatic blood lactate test meter (Lactate Pro; Arkray) and glucose test meter (Dexter ZII; Bayer), respectively.

### Histological Analyses

Formalin-fixed, paraffin-embedded serial sections were used for histological analyses. Hematoxylin and eosin–stained sections were used for histopathological analysis to identify tumor tissues. The immunohistochemical analysis was performed with antibody to CD45 (BD Biosciences) to determine whether the tumor tissues originated from leukocytes, and subsequently with antibodies to B220 (BD Biosciences) and CD3 (Santa Cruz) to determine whether the tumor tissues were of B-cell or T-cell origin, respectively.

### Measurement of ROS Production in Mitochondria

ROS generation was detected with the mitochondrial superoxide indicator MitoSOX-Red (Life Technologies). Cells were incubated with 1 µM MitoSOX-Red for 15 min at 37°C in phosphate-buffered saline (PBS), washed twice with PBS, and then immediately analyzed with a FACScan flow cytometer (Becton Dickinson).

### Statistical Analysis

Data were analyzed by Dunnett’s test or one-way ANOVA followed by Dunnett’s post test. Kaplan–Meier curves were assessed with the log-rank test. Values with *P*<0.05 were considered significant.

## Results

### Mitochondrial Respiration Defects in mtDNA Mutator Mice with a B6J Nuclear Background

**Figure 1:**
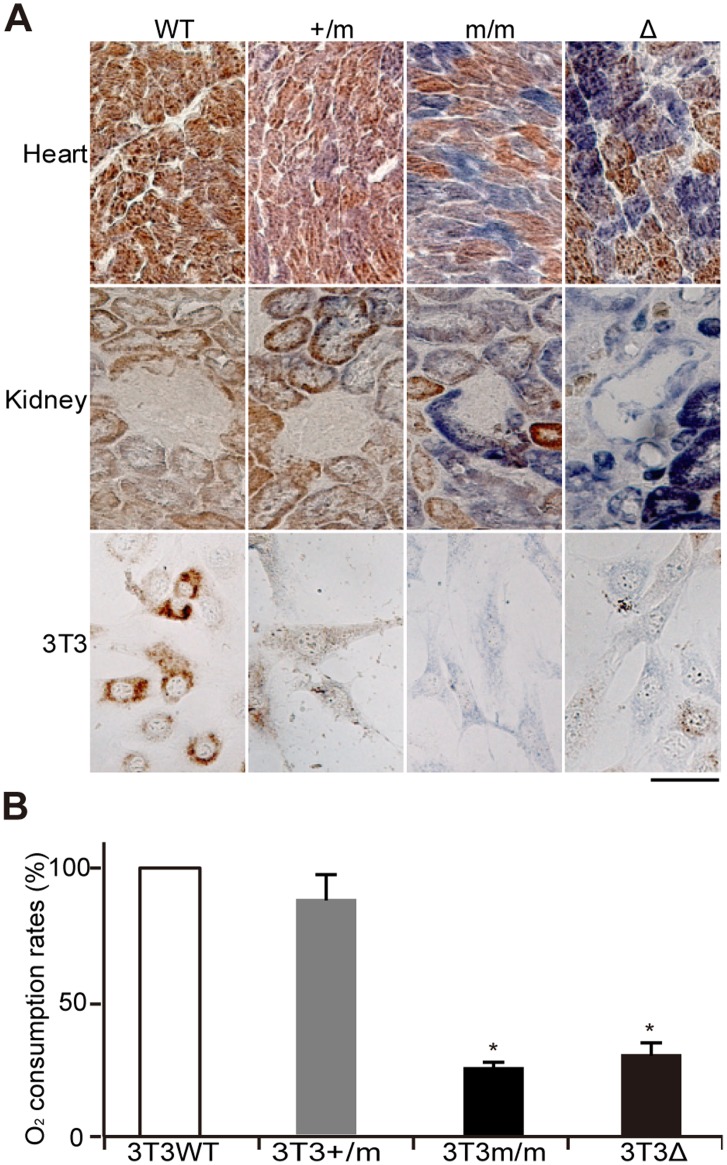
Comparison of mitochondrial respiratory function in mtDNA mutator mice and mito-miceΔ. WT, wild-type mice; +/m, heterozygous mutator mice; m/m, homozygous mutator mice; Δ, mito-miceΔ. (A) Histochemical analysis of mitochondrial respiratory enzyme activities in the heart, kidney, and 3T3 cells. Tissue sections and 3T3 cells were stained for cytochrome *c* oxidase (COX) (brown) and succinate dehydrogenase (SDH) (blue). Cells that had lost COX activity were detected as a blue colour. The proportions of ΔmtDNA in the heart, kidney tissues, and 3T3 cells from mito-miceΔ were 82.6%, 79.1%, and 73.8%, respectively. A scale bar, 50 µm. (B) Measurement of O_2_ consumption rates in 3T3 cells. Data are represented as mean values with SD (n = 3). *P<0.05 compared with wild-type mice.

We generated heterozygous (+/m) and homozygous (m/m) mtDNA mutator mice with a B6J nuclear background (see Materials and Methods), and examined whether mtDNA mutator mice with a B6J nuclear background also express respiration defects. Young (10-month-old) mice were used for examination of their mitochondrial respiratory function. Age-matched normal B6J mice and mito-miceΔ with a B6J nuclear background were used as positive and negative controls, respectively. Histochemical analysis of mitochondrial cytochrome *c* oxidase (COX) activity in mouse tissues showed reduced COX activity in homozygous m/m mutator mice and mito-miceΔ, and mild COX defects in heterozygous +/m mutator mice (Fig. 1A).

For quantitative estimation of overall mitochondrial respiratory function, we isolated immortalized 3T3 cell lines from mouse embryonic fibroblasts (MEFs) by using the 3T3 protocol [18], [19]. Immortalized 3T3 cell lines obtained from m/m mutator mice (3T3m/m) and mito-miceΔ (3T3Δ) showed a similar reduction in both COX activity (Fig. 1A) and O_2_ consumption rates compared to controls (Fig. 1B). Thus, m/m mutator mice express notable respiration defects, even when they share B6J nuclear genetic background with that of mito-miceΔ.

### Cotransfer of mtDNA and Respiration Defects from m/m Mice into ρ^0^ Mouse B82 Cells

We then addressed whether respiration defects found in mtDNA mutator mice (Fig. 1) are caused by abnormalities accumulated in mtDNA or in nuclear DNA. It is possible that the genetic complementation activity present in mammalian mitochondria [10], [15], [16] prevents tissues from expressing respiration defects caused by the accumulated random mutations in mtDNA. Moreover, considering that respiratory functions are controlled by both mtDNA and nuclear DNA, it is still possible that abnormalities in nuclear DNA are responsible for respiration defects, even though mtDNA mutator mice are prone to accumulate various somatic mutations in mtDNA [6], [7], [21]–[23].

To examine this possibility, we transferred mitochondria from the platelets of +/m and m/m mutator mice (10 months old) into ρ^0^ mouse B82 cells by their fusion with the platelets. Selection medium without uridine and pyruvate excluded the unfused ρ^0^ mouse B82 cells, and allowed exclusive growth of B82mt+/m and B82mtm/m transmitochondrial cybrids, which share the B82 nuclear background but carry mtDNA from +/m and m/m mice, respectively (Table S1). As positive controls, we used B82mtWT transmitochondrial cybrids, which were obtained by the fusion of ρ^0^ mouse B82 cells with the platelets from age-matched wild-type (WT) B6J mice (Table S1).

**Figure 2:**
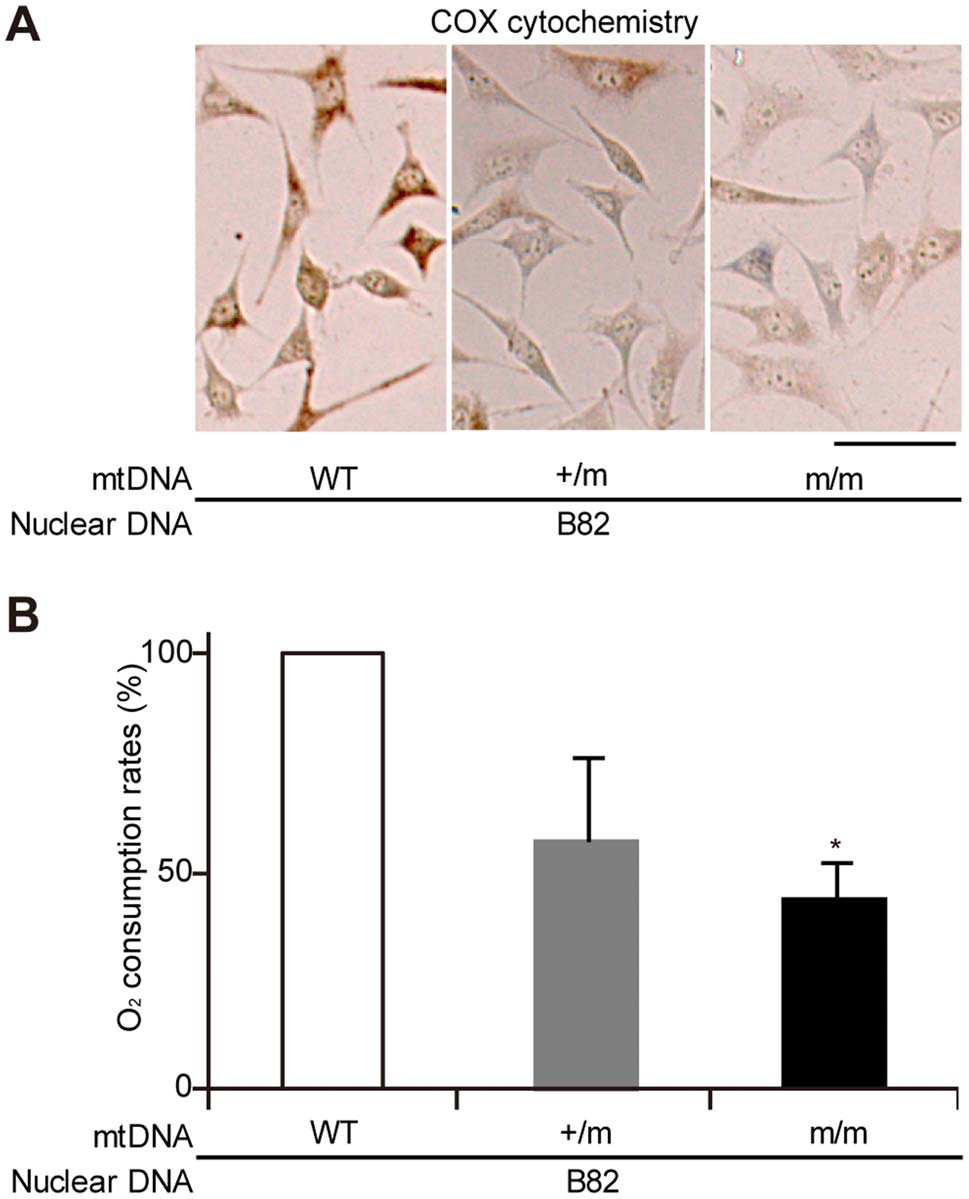
Cotransfer of mtDNA and respiration defects from mtDNA mutator mice into ρ^0^ mouse B82 cells. WT, wild-type mice; +/m, heterozygous mutator mice; m/m, homozygous mutator mice. (A) Cytochemical analysis of mitochondrial respiratory enzyme activities in trans-mitochondrial cybrids. Cells that had lost COX activity were detected as a blue colour. A scale bar, 50 µm. (B) Measurement of O_2_ consumption rates. Data are represented as mean values with SD (n = 3). *P<0.05 compared with wild-type mice.

All isolated transmitochondrial cybrids were cultivated for 2 months to obtain a sufficient number of cells to estimate O_2_ consumption rates. COX activity (Fig. 2A) and O_2_ consumption rates (Fig. 2B) were reduced significantly in B82mtm/m cybrids compared to controls. These results suggest that mitochondrial respiration defects were co-transferred with the mtDNA from m/m mice into ρ^0^ mouse B82 cells, providing convincing evidence that respiration defects expressed in mtDNA mutator mice are due to mtDNA abnormalities created by the deficient proofreading function of mtDNA polymerase. Moreover, these observations also suggest that the transferred respiration defects were not restored during the prolonged 2-month long cultivation of B82mtm/m cybrids, even under conditions where the nuclear genome of the cybrids was derived from B82 cells possessing mtDNA polymerase with a normal proofreading function. Therefore, mtDNA abnormalities are furthermore transferable to following generations of the cybrids, and would correspond to mtDNA mutations.

### Sequence and Southern Blot Analyses of mtDNA from m/m Mice

**Figure 3:**
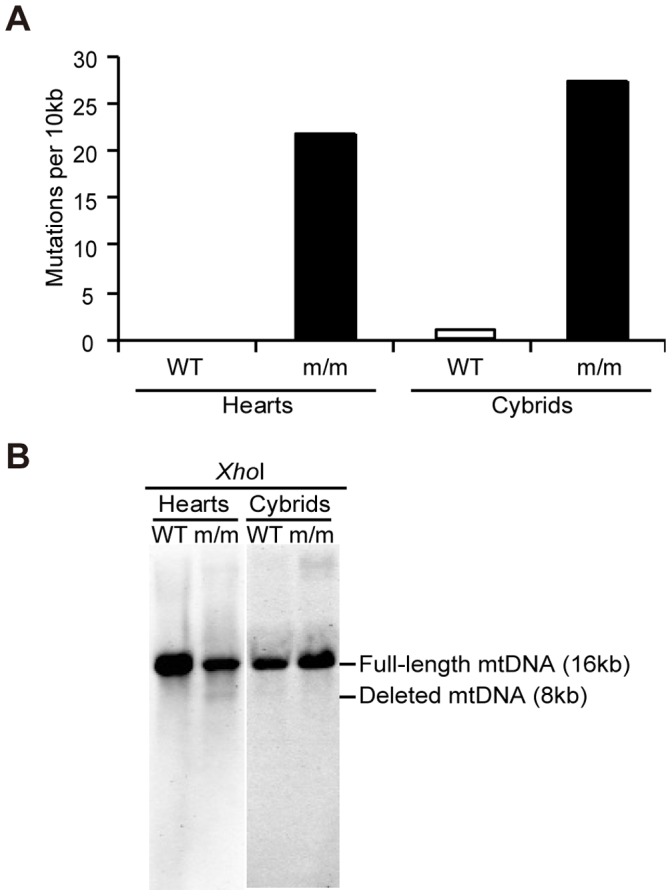
Sequence and Southern blot analyses of mtDNA from mtDNA mutator mice. Total DNA was prepared from the hearts of a 10-month-old wild-type mouse (WT) and an age matched homozygous mutator mouse (m/m), and from the trans-mitochondrial cybrids carrying mtDNA from platelets of a 10-month-old wild-type mouse and age-matched m/m mutator mouse, respectively. (A) Sequence analysis of 517 bp-fragments of the *COX1* gene. Numbers of somatic mutations in mtDNA of the hearts from a wild-type mouse and an m/m mouse, and of the cybrids with mtDNA of a wild-type and an m/m mouse were counted. (B) Southern blot analysis of *Xho*I-digested mtDNA. Lanes 1 and 2 represent the hearts of a WT and an m/m mouse, respectively. Lanes 3 and 4 represent the cybrids with mtDNA of a wild-type and an m/m mouse, respectively.

To examine whether the mtDNA abnormalities correspond to point mutations or deletion mutations of mtDNA, we carried out sequence analysis (Fig. 3A) and Southern blot analysis (Fig. 3B) of mtDNA prepared from the heart of an m/m mouse (10 months old) and B82mtm/m cybrids. The heart of an age-matched B6J (wild-type) mouse and B82mtWT cybrids were used as controls.

Sequence analysis of fifty clones of a part of *COX1* gene revealed that significant amounts of point mutations are accumulated in mtDNA from the heart of an m/m mutator mouse, while no mutations were found in the heart of an age-matched B6J mouse (Fig. 3A). Preferential accumulation of somatic point mutations in mtDNA was also observed in B82mtm/m cybrids, when we compared mtDNA sequence between B82mtm/m and B82mtWT cybrids (Fig. 3A). Therefore, enhanced accumulation of the mtDNA point mutations would partly be responsible for the respiration defects found in tissues of m/m mutator mice (Fig. 1) and in B82mtm/m cybrids (Fig. 2).

Southern blot analysis also showed that the heart of an m/m mouse possessed deleted mtDNA fragments (about 8 kbp) as well as mtDNA with normal sizes (about 16 kbp). However, B82mtm/m cybrids did not possess detectable amounts of the deleted mtDNA fragments (Fig. 3B). These observations suggest that the deleted mtDNA produced in the tissues of the m/m mouse can partly be responsible for the respiration defects (Fig. 1), but are not able to replicate and confer respiration defects in B82mtm/m cybrids (Fig. 2). Thus, the deleted products would correspond to the linear mtDNA fragments newly created by the mutated polymerase gamma of m/m mice [22], but not to mtDNA with deletion mutations.

### Lifespan and Premature Aging Phenotypes

Next, we investigated whether homozygous m/m mutator mice carrying a B6J nuclear background also express premature aging phenotypes. To examine this idea we started the experiments using 6-month-old mice: 19 wild-type B6J mice, 71 mtDNA mutator mice (39+/m and 32 m/m), and 25 mito-miceΔ^2.0–60.8^ with 2.0–60.8% ΔmtDNA in their tails at 4 weeks after the birth.

**Figure 4:**
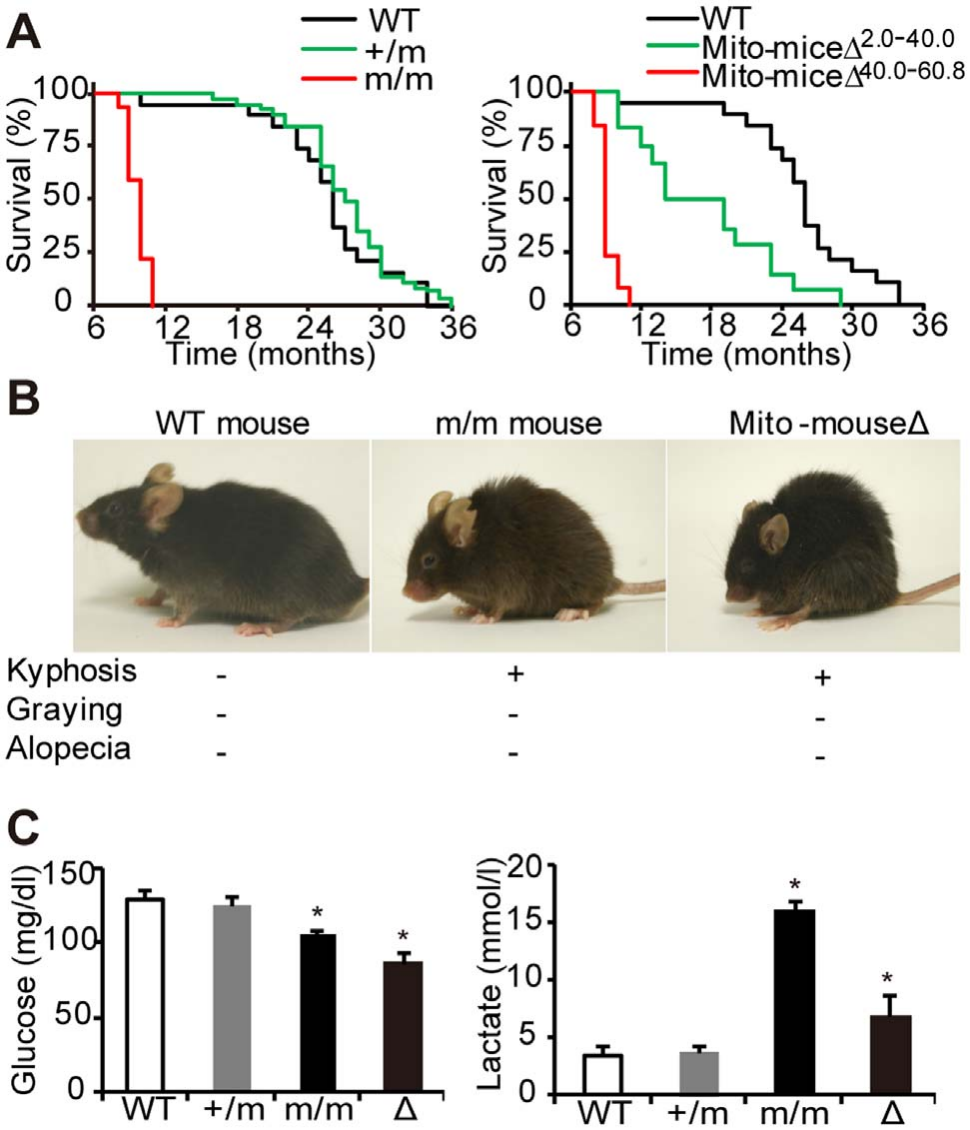
Comparison of the phenotypes observed in mtDNA mutator mice and mito-miceΔ. (A) Kaplan–Meier survival curves of mtDNA mutator mice and mito-miceΔ. Median survival times of wild-type (WT) mice (n = 19), +/m mice (n = 39), m/m mice (n = 32), mito-miceΔ^2.0–40.0^ (n = 12), and mito-miceΔ^40.0–60.8^ (n = 13) were 26, 27, 10, 17, and 9 months, respectively. (B) WT mouse, m/m mouse, and mito-mouseΔ at 9 months of age. Kyphosis was observed in homozygous m/m mutator mice and mito-miceΔ, while hair graying and hair loss (alopecia) were not observed. (C) Estimation of blood glucose and blood lactate levels in mutator mice and mito-miceΔ^40–60.8^ at 9 months of age. WT, wild-type mice; +/m, heterozygous mutator mice; m/m, homozygous mutator mice; Δ, mito-miceΔ. Data are represented as mean values with SD (n = 3). *P<0.05 compared with wild-type mice.

Median survival times of wild-type, +/m, and m/m mutator mice were 26, 27, and 10 months, respectively (Fig. 4A), showing that homozygous m/m mutator mice have a much shorter lifespan than controls, even under a B6J nuclear background. Median survival times of 10 months for our m/m mutator mice with a B6J nuclear background is slightly shorter than the 11 months [6] and 14 months [7] of other m/m mutator mice (Table S2), probably due to the differences in nuclear background and/or conditions for feeding and maintenance.

When the proportions of ΔmtDNA in tails were restricted to higher levels (40.0%–60.8%), 13 mito-miceΔ^40.0–60.8^ possessing 40.0%–60.8% ΔmtDNA in their tails had a very short lifespan (9 months; Fig. 4A) comparable to that of m/m mutator mice (10 months; Fig. 4A). Thus, damages of mtDNA would be very similar between m/m mice and mito-miceΔ^40.0–60.8^. Moreover, all 13 mito-miceΔ^40.0–60.8^ showed kyphosis (Fig. 4B), which has been observed in m/m mutator mice [6], [7] and confirmed in this study to be expressed in m/m mutator mice with a B6J nuclear background (Figs. 4B and Table S2). However, alopecia, which has been reported in m/m mutator mice as a typical premature aging phenotype [6], [7], was not observed in our m/m mutator mice and mito-miceΔ^40.0–60.8^ sharing the same B6J nuclear background (Fig. 4B). The absence of alopecia in both our m/m mutator mice and mito-mceΔ^40.0–60.8^ suggest that the apparent discrepancy in the expression of premature aging phenotypes that were observed exclusively in mtDNA mutator mice [6], [7], but not in mito-miceΔ [9], [10] might partly be related to slight differences in their nuclear genetic background (Table S2).

Moreover, both the homozygous m/m mutator mice with a B6J nuclear background and mito-mceΔ^40.0–60.8^ had low blood glucose and high blood lactate levels, while heterozygous +/m mutator mice were normal in their levels (Fig.4C). These results are consistent with the findings of previous studies [9], [24], [25] with the exception that m/m mutator mice with a B6J nuclear background have low blood glucose levels (Fig. 4C). These observations suggest that homozygous m/m mutator mice have potential as a model for the study of mitochondrial diseases as well as of aging. However, our m/m mutator mice and mito-mceΔ^40.0–60.8^ sharing the B6J nuclear background also showed different phenotypes associated with diseases. For example, m/m mutator mice expressed significant increase in the amounts of blood lactate levels (Fig. 4C). On the contrary, mito-miceΔ exclusively had enlarged kidneys with a granulated surface with renal failures [10]. Considering that mito-miceΔ accumulated the same ΔmtDNA with age, and that m/m mutator mice accumulated mtDNA with various somatic mutations with age, the difference of mutations in mtDNA may partly be responsible for the difference of their phenotypes.

### Tumor Formation Frequencies of +/m Mice

**Figure 5:**
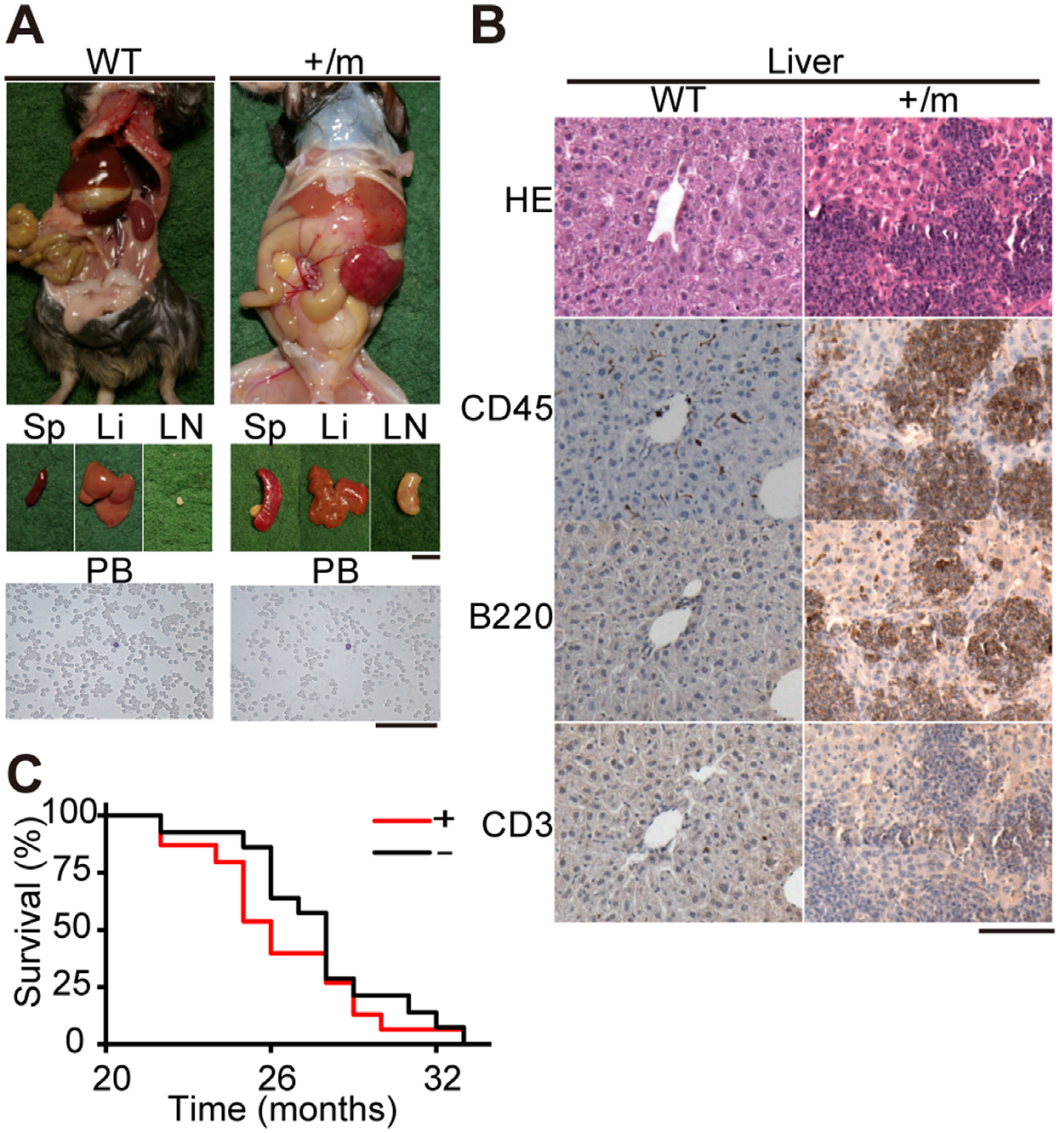
B-cell lymphoma formation in the tissues of aged +/m mutator mice. WT, wild-type mice; +/m, heterozygous mutator mice. (A) Gross necropsy of euthanized moribund mice (upper panels), tissues (middle panels), and smear samples of peripheral blood stained with Giemsa (lower panels). Left and right panels represent a euthanized moribund wild-type (WT) mouse without tumors and a euthanized moribund +/m mouse with tumors (+/m mouse-4; see Table S2), respectively. Giemsa-stained preparations show the absence of leukemic cells in the peripheral blood of both wild-type and +/m mice. Sp, spleen; Li, liver; LN, lymph node; PB, peripheral blood. Scale bars represent 1 cm (middle panels) and 50 µm (lower panels). (B) Histological analyses of serial sections of the liver to identify B-cell lymphoma. Hematoxylin and eosin (HE) staining to show tumor formation; CD45, immunohistochemistry using antibody to CD45 to detect leukocytes; B220, immunohistochemistry using antibody to B220 to detect B cells; CD3, immunohistochemistry using antibody to CD3 to detect T cells. The tissues of WT mice have a normal structure (left), whereas those of +/m mice show of the development of B-cell lymphoma, because they stained positively with CD45 and B220, but not with CD3 (right). A scale bar, 50 µm. (C) Kaplan–Meier survival curves of +/m mice with or without lymphoma. Median survival times of +/m mice with lymphoma (n = 15) and +/m mice without lymphoma (n = 14) were 26 and 28 months, respectively. +, +/m mice with lymphoma; −, +/m mice without lymphoma; *P* = 0.362.

Although heterozygous +/m mutator mice showed mild respiration defects (Fig. 1), they had a normal lifespan (Fig. 4A) that was comparable to a lifespan of wild-type mice. These results are consistent with the findings of a previous study [26] that showed that median survival times of +/m mice did not change substantially from those of wild-type mice. However, gross necropsy of all dead or euthanized moribund mice revealed that 15 of 29+/m mutator mice (52%) had macroscopic tumor-like abnormalities in spleen, liver and/or lymph nodes (Table S3 and Fig. 5A). By comparison, only 2 of 12 wild-type mice (17%) and none of the 32 m/m mutator mice showed tumor-like abnormalities (Table S3).

Histological analyses revealed that all abnormal tissues were hematopoietic neoplasms and were positive for the pan-leukocyte marker CD45 (Table S3 and Fig. 5B). Since there was no increase in the number of leukemic cells in the peripheral blood of +/m mice compared with wild-type mice (Fig. 5A), these hematological neoplasms most likely consisted of lymphoma cells. All tumors were of B-cell origin because they expressed the B-cell marker B220 (Fig. 5B), and arose in the spleen, liver, lung, and/or lymph nodes (Table S3 and Fig. 5A). These data indicated that compared to wild-type mice, the +/m mutator mice were more prone to B-cell lymphoma development.

The median survival times of +/m mutator mice with and without B-cell lymphoma were 26 and 28 months, respectively (Fig. 5C). The shorter lifespan of +/m mutator mice with B-cell lymphoma compared with that of +/m mutator mice without B-cell lymphoma is most likely partly the result of B-cell lymphoma development.

### Estimation of ROS in Bone Marrows of +/m Mice with and without B-cell Lymphomas

**Figure 6:**
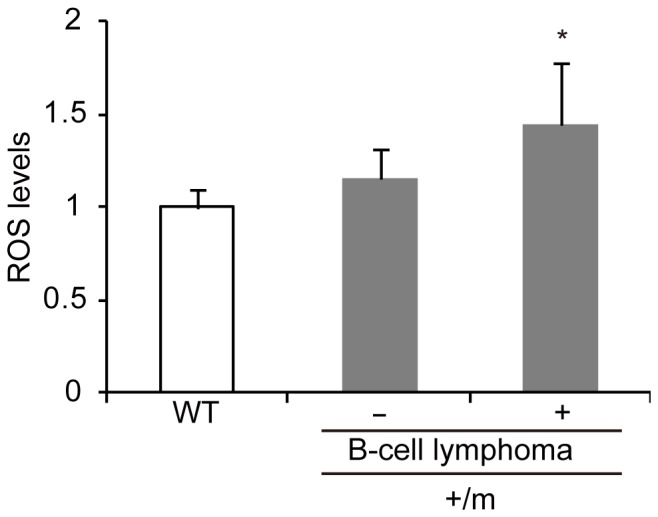
Estimation of mitochondrial ROS levels in bone marrow cells of +/m mice with and without B-cell lymphoma. WT, wild-type mice; +/m, heterozygous mutator mice; −, mice without lymphoma; +, mice with lymphoma. Relative mitochondrial superoxide levels in +/m mice without B-cell lymphoma and +/m mice with B-cell lymphoma were expressed as mean ﬂuorescence intensity after treatment with MitoSOX Red (Life Technologies). Data are represented as mean values with SD (n = 5). *P<0.05 compared with wild-type mice.

Our previous report showed that aged mito-miceND6^M^ carrying an mtDNA point mutation G13997A in the *ND6* gene frequently developed B cell-lymphomas [14]. Because bone marrow cells of mito-miceND6^M^ overproduce ROS [14], the overproduction of ROS in bone marrow cells of +/m mutator mice might be crucial for the development of B-cell lymphoma (Table S3). To examine this idea, we compared the amount of mitochondrial ROS in the bone marrow cells of wild-type mice with that of +/m mutator mice (20–25 months old). An increase in the amount of mitochondrial ROS was observed only in +/m mutator mice with B-cell lymphomas (Fig. 6). It is therefore likely that the overproduction of ROS in bone marrow cells of +/m mutator mice plays an important part in the formation of B-cell lymphoma.

## Discussion

By generating mtDNA mutator mice with the same B6J nuclear background as that of mito-miceΔ, we can provide an answer to the question of why premature aging phenotypes are exclusively observed in homozygous m/m mutator mice but not in transmitochondrial mito- miceΔ, even though they both express significant respiration defects. We showed that the significant respiration defects and high frequency of mtDNA mutations were expressed in m/m mutator mice generated here, and can be transferred together with the transfer of mtDNA from the platelets of the m/m mutator mice into ρ^0^ B82 cells (Figs. 1, 2, 3). Thus, our mutator mice also express respiration defects, even under a B6J nuclear background, and respiration defects found in mutator mice are caused by abnormalities in their mtDNA. However, the m/m mutator mice with a B6J nuclear background did not express the premature aging phenotypes of graying and alopecia, while they did express kyphosis and had a short lifespan (Fig. 4). Similar phenotypes were observed in mito-miceΔ^40.0–60.8^, when the proportions of ΔmtDNA were restricted to higher levels (Fig. 4). Therefore, the expression patterns of premature aging phenotypes of m/m mutator mice with a B6J nuclear background are very similar to that of mito-miceΔ carrying predominant amounts of ΔmtDNA in that they both express kyphosis and have a short lifespan, but do not express graying and alopecia. These observations suggest that the apparent discrepancy in the presence and absence of premature aging phenotypes in mutator mice and mito-mice from previous studies is partly the result of differences in their nuclear genetic background.

Heterozygous +/m mutator mice showed only slight respiration defects (Figs. 1 and 2) and had a normal lifespan comparable to that of wild-type mice (Fig. 4A), which are findings consistent with a previous publication [26]. However, this study provided the new evidence that +/m mutator mice with a B6J nuclear background frequently develop age-associated B-cell lymphomas. The +/m mutator mice developed no tumors other than B-cell lymphomas (Table S3), despite the presence of mtDNA abnormalities in all the tissues. Considering that 17% of the wild-type mice formed B-cell lymphoma but no other tumors (Table S3), one answer to this tissue-specific tumor development in +/m mice is that the nuclear background of the B6J mice used in this study made them prone to the development of B-cell lymphomas. In support of this notion, it has been reported that the nuclear genetic background affects the spectrum of tumors that develop in mice [27]–[30].

With respect to the mechanism underlying the development of B-cell lymphoma in +/m mice, the overproduction of ROS in bone marrow cells may be related, because oxidative stress induces various types of cellular damages that can lead to genetic instability and subsequent tumor development [31]. However, it has been reported that tissues and cells from mtDNA mutator mice do not overproduce ROS [7], [8], [32]. Our study also showed that bone marrow cells in +/m mice do not overproduce mitochondrial ROS (Fig. 6). In contrast, bone marrow cells from +/m mice carrying B-cell lymphomas exclusively overproduced mitochondrial ROS (Fig. 6). It is therefore possible that a population of bone marrow cells was induced to overproduce ROS as the results of B-cell lymphoma development. It is also possible that a small population of bone marrow cells accumulates specific mtDNA abnormalities that, by chance, induce ROS overproduction resulting in the development of B-cell lymphoma. The latter idea is supported by our recent findings that B-cell lymphomas developed preferentially in transmitochondrial mito-miceND6^M^ carrying a ROS-inducing mtDNA mutation [14] but not in transmitochondrial mito-miceCOI^M^ carrying an mtDNA point mutation that does not induce ROS overproduction [14]. Taken together, these observations suggest that mtDNA abnormalities in +/m mice do not accelerate aging (Fig. 4A), but preferentially induce B-cell lymphoma development (Table S3).

Because our previous studies [10], [15], [16] demonstrated the presence of inter-mitochondrial interactions and the resultant genetic complementation that occurs in mammalian mitochondria, it is possible that accumulated random mutations in mtDNA complemented each other and fail to induce the respiration defects found in mtDNA mutator mice. However, this study provided convincing evidence that respiration defects can be transferred together with mtDNA from mtDNA mutator mice into ρ^0^ mouse B82 cells (Fig. 2). These findings suggest that respiration defects in mtDNA mutator mice (Fig. 1) are caused by abnormalities in their mtDNA. One explanation of why random mutations in the mtDNA of mtDNA mutator mice induce respiration defects in the presence of mitochondrial genetic complementation is that the extremely high frequency of somatic mutations in mtDNA causes instability of the large mitochondrial respiration complexes thereby resulting in respiration defects, even when somatic mutations occur at random sites [23]. This idea could be examined by complete sequence analysis of mtDNA in tissues of m/m mice.

## Supporting Information

Table S1 **Isolation of the trans-mitochondrial cybrids. ^a^**B82 cells are fibrosarcomas derived from the L929 fibroblast cell line (C3H/An mouse strain), and ρ^0^ B82 cells without their own mtDNA were isolated in our previous report (9). **^b^**UP- represent the selection medium without uridine and pyruvate to exclude unfused ρ^0^ B82 cells.
(DOC)

Table S2 **Characterization of the mice used in the previous studies and this study. ^a^** A B6J strain used in this study corresponds to a B6JJcl strain generated by sibling mating more than 40 times in CLEA Japan (Jcl). **^b^** Expression of a hair graying phenotype is not detectable in this strain because of its phenotypic expression of white hair color [6]. **^c^** Alopecia was observed in m/m mice with B6 strain nuclear genome [7] and in m/m mice with 129R1/B6 strain nuclear genome [6], but not in m/m mice with B6JJcl nuclear genome generated in this study. Since nuclear genomes are very close between B6 strain used in the previous study [7] and B6JJcl strain used in this study, variability of nuclear genome may not be responsible for the lack of alopecia in our m/m mice. On the contrary, this study also showed that m/m mice as well as mito-miceΔ sharing the same B6JJcl nuclear genetic background and feeding conditions did not express alopecia (Fig. 4), suggesting that slight variability of nuclear genome between B6 and B6JJcl mice and/or different conditions for feeding and maintenance may due at least in part to the discrepancy that the alopecia was not observed in m/m mice of this study.
(DOC)

Table S3 **Frequencies of lymphoma in dead or moribund mice.** ^a^ Individual codes were allocated in order of death.
(DOC)
