# Supplementary material for: Mitochondrial DNA Mutations in Mutator Mice Confer Respiration Defects and B-Cell Lymphoma Development
Source: PLoS One. 2013 Feb 13;8(2):e55789. doi: 10.1371/journal.pone.0055789 (PMC3572082; doi:10.1371/journal.pone.0055789)
Supplement: Table S1 — Isolation of the trans-mitochondrial cybrids. aB82 cells are fibrosarcomas derived from the L929 fibroblast cell line (C3H/An mouse strain), and ρ0 B82 cells without their own mtDNA were isolated in our previous report (9). bUP- represent the selection medium without uridine and pyruvate to exclude unfused ρ0 B82 cells. (DOC) [file pone.0055789.s001.doc]

**Table S1. Isolation of the trans-mitochondrial cybrids**

|  |  | Fusion combination |  |  |
| --- | --- | --- | --- | --- |
| Cells |  | Nucleara donors X mtDNA donors |  | Selection |
| Nuclear donors=mtDNA recipients |  |  |  |  |
| ρ^0^ B82 cells**^a^** |  |  |  |  |
| mtDNA donors |  |  |  |  |
| platelets (WT mice) |  |  |  |  |
| platelets (+/m mice) |  |  |  |  |
| platelets (m/m mice) |  |  |  |  |
| Cybrids |  |  |  |  |
| B82mtWT |  | ρ^0^ B82 cells X platelets (WT mice) |  | UP-**^b^** |
| B82mt+/m |  | ρ^0^ B82 cells X platelets (+/m mice) |  | UP- |
| B82mtm/m |  | ρ^0^ B82 cells X platelets (m/m mice) |  | UP- |
