# Supplementary material for: Mitochondrial DNA Mutations in Mutator Mice Confer Respiration Defects and B-Cell Lymphoma Development
Source: PLoS One. 2013 Feb 13;8(2):e55789. doi: 10.1371/journal.pone.0055789 (PMC3572082; doi:10.1371/journal.pone.0055789)
Supplement: Table S2 — Characterization of the mice used in the previous studies and this study. a A B6J strain used in this study corresponds to a B6JJcl strain generated by sibling mating more than 40 times in CLEA Japan (Jcl). b Expression of a hair graying phenotype is not detectable in this strain because of its phenotypic expression of white hair color [6]. c Alopecia was observed in m/m mice with B6 strain nuclear genome [7] and in m/m mice with 129R1/B6 strain nuclear genome [6], but not in m/m mice with B6JJcl nuclear genome generated in this study. Since nuclear genomes are very close between B6 strain used in the previous study [7] and B6JJcl strain used in this study, variability of nuclear genome may not be responsible for the lack of alopecia in our m/m mice. On the contrary, this study also showed that m/m mice as well as mito-miceΔ sharing the same B6JJcl nuclear genetic background and feeding conditions did not express alopecia (Fig. 4), suggesting that slight variability of nuclear genome between B6 and B6JJcl mice and/or different conditions for feeding and maintenance may due at least in part to the discrepancy that the alopecia was not observed in m/m mice of this study. (DOC) [file pone.0055789.s002.doc]

**Table S2. Characterization of the mice used in the previous studies and this study**

|  | m/m mutator mice (Ref. 6) | m/m mutator mice (Ref. 7) | m/m mutator mice (this study) | mito-miceΔ^40.0-60.8^ (this study) |
| --- | --- | --- | --- | --- |
|  |
| Strain | 129;B6 | B6 | B6J^a^ | B6J^a^ |
| Lifespan (months) | 11 | 14 | 10 | 9 |
| Kyphosis | + | + | + | + |
| Hair graying | Not detectable^b^ | + | - | - |
| Alopecia^c^ | + | + | - | - |
