# Supplementary material for: Mitochondrial DNA Mutations in Mutator Mice Confer Respiration Defects and B-Cell Lymphoma Development
Source: PLoS One. 2013 Feb 13;8(2):e55789. doi: 10.1371/journal.pone.0055789 (PMC3572082; doi:10.1371/journal.pone.0055789)
Supplement: Table S3 — Frequencies of lymphoma in dead or moribund mice. a Individual codes were allocated in order of death. (DOC) [file pone.0055789.s003.doc]

**Table S3. Frequencies of lymphoma in dead or moribund mice**

|  |  | No. of mice with lymphoma (Individual code ^a^) |  | Tissues with tumor | | | |  | Histological analyses | | | |  |
| --- | --- | --- | --- | --- | --- | --- | --- | --- | --- | --- | --- | --- | --- |
| mice | Total no. of mice |  | Life span (months) | Spleen | Liver | Lung | Lymph node |  | HE | CD45 | B220 | CD3 | Cell lineage |
| +/+ | 12 | 2 |  |  |  |  |  |  |  |  |  |  |  |
|  |  | (5) | 25 | + |  |  | + |  | Lymphoma | + | + | - | B cell |
|  |  | (8) | 24 | + |  |  | + |  | Lymphoma | + | + | - | B cell |
|  |  |  |  |  |  |  |  |  |  |  |  |  |  |
| +/m | 29 | 15 |  |  |  |  |  |  |  |  |  |  |  |
|  |  | (1) | 29 | + |  |  | + |  | Lymphoma | + | + | - | B cell |
|  |  | (2) | 30 | + |  |  |  |  | Lymphoma | + | + | - | B cell |
|  |  | (3) | 25 | + | + |  | + |  | Lymphoma | + | + | - | B cell |
|  |  | (4) | 28 | + | + |  | + |  | Lymphoma | + | + | - | B cell |
|  |  | (5) | 28 | + | + |  | + |  | Lymphoma | + | + | - | B cell |
|  |  | (6) | 25 | + |  |  | + |  | Lymphoma | + | + | - | B cell |
|  |  | (8) | 26 | + | + |  | + |  | Lymphoma | + | + | - | B cell |
|  |  | (10) | 24 | + | + |  | + |  | Lymphoma | + | + | - | B cell |
|  |  | (11) | 25 | + | + |  | + |  | Lymphoma | + | + | - | B cell |
|  |  | (12) | 29 | + | + |  | + |  | Lymphoma | + | + | - | B cell |
|  |  | (13) | 25 | + | + | + |  |  | Lymphoma | + | + | - | B cell |
|  |  | (23) | 22 | + | + |  | + |  | Lymphoma | + | + | - | B cell |
|  |  | (24) | 22 | + |  |  | + |  | Lymphoma | + | + | - | B cell |
|  |  | (25) | 33 | + |  | + |  |  | Lymphoma | + | + | - | B cell |
|  |  | (29) | 26 | + |  |  | + |  | Lymphoma | + | + | - | B cell |
|  |  |  |  |  |  |  |  |  |  |  |  |  |  |
| m/m | 32 | 0 |  |  |  |  |  |  |  |  |  |  |  |
